# Real-Time Eyeblink Detector and Eye State Classifier for Virtual Reality (VR) Headsets (Head-Mounted Displays, HMDs)

**DOI:** 10.3390/s19051121

**Published:** 2019-03-05

**Authors:** Nassr Alsaeedi, Dieter Wloka

**Affiliations:** Department of Electrical Engineering and Computer Science, University of Kassel, 34127 Kassel, Germany; dwloka@uni-kassel.de

**Keywords:** eyeblink, real-time eye blink detection, eye state classification, head-mounted displays (HMD’s), virtual reality, motion vector analysis, cross-correlation

## Abstract

The aim of the study is to develop a real-time eyeblink detection algorithm that can detect eyeblinks during the closing phase for a virtual reality headset (VR headset) and accordingly classify the eye’s current state (open or closed). The proposed method utilises analysis of a motion vector for detecting eyelid closure, and a Haar cascade classifier (HCC) for localising the eye in the captured frame. When the downward motion vector (DMV) is detected, a cross-correlation between the current region of interest (eye in the current frame) and a template image for an open eye is used for verifying eyelid closure. A finite state machine is used for decision making regarding eyeblink occurrence and tracking the eye state in a real-time video stream. The main contributions of this study are, first, the ability of the proposed algorithm to detect eyeblinks during the closing or the pause phases before the occurrence of the reopening phase of the eyeblink. Second, realising the proposed approach by implementing a valid real-time eyeblink detection sensor for a VR headset based on a real case scenario. The sensor is used in the ongoing study that we are conducting. The performance of the proposed method was 83.9% for accuracy, 91.8% for precision and 90.40% for the recall. The processing time for each frame took approximately 11 milliseconds. Additionally, we present a new dataset for non-frontal eye monitoring configuration for eyeblink tracking inside a VR headset. The data annotations are also included, such that the dataset can be used for method validation and performance evaluation in future studies.

## 1. Introduction

Recently, eyeblink detection has been the subject of significant attention in the human–computer interaction domain, with a considerable amount of research being conducted utilising this method, for instance, in the automobile accident prevention domain for monitoring driver fatigue and drowsiness [1,2,3], the disabled assistant domain [4] and in the healthcare domain, such as detecting computer vision syndrome [5]. Different approaches have been adopted in these domains to detect eyeblinks. These involve employing several techniques to analyse the captured frames, such as optical flow [6], template matching [7] and contour analysis [8]. These techniques generate an eyeblink waveform after analysing the captured frames, which is then post-processed to detect eyeblinks. Furthermore, these methods use a frontal-monitoring setup to observe the complete face of the participant. In our study, we focus on a video-based eye monitoring approach to eyeblink detection.

The issue of detecting eyeblinks for a VR headset has other requirements when compared to the above described methods in the mentioned domains. First, the participant is wearing a VR headset, which partially occludes the face and completely covers the eyes. Second, the monitoring camera is mounted inside the VR headset, which makes the lighting conditions of this case different from the complete face monitoring setup. Additionally, a non-frontal monitoring setup is employed to monitor the eye to avoid the occlusion of the VR headset’s display. These points make the available eyeblink datasets in the community inappropriate to train and validate our proposed approach.

A few studies have attempted to address the eyeblink detection problem in virtual reality (VR). However, at the time of conducting this study, eyeblink tracking is not standard in the current consumer versions of VR headsets, like HTC-Vive, Oculus and PlayStation, but there are many VR applications that can utilise real-time eyeblink tracking. For instance, avatars’ eyeblinks can be animated to make them more lifelike during social interaction in Immersive Virtual Environments (IVEs). Another application is repositioning and reorienting the participant imperceptibly in an IVE to tackle the locomotion issue in VR. Moreover, combining information about a participant’s head motion patterns and eyeblink frequency can be deployed to identify different types of high-level cognitive activities, such as, reading, talking or performing tasks, that require high cognitive effort [9]. Additionally, the possibility of exposure to dry eye syndrome (eye fatigue, dryness, soreness, red-eye and headache) due to lengthy VR sessions can be monitored and thus, participant fatigue can be detected [5].

The main objective of this study is to implement a real-time eyeblink detector able to detect eyeblinks during the closing or pause phases of the eyeblink, and before the reopening phase. This is crucial before future proposed work can be embarked upon, which is to reposition and reorient the participant in an imperceptible way during the eyeblink. The capturing device and the processing algorithm should both be fast enough to fulfil the task. The next section will explain some eyeblink terminology before a review of the related research is presented.

### Eyeblinks and Blinking Kinematics

**Figure 1:**
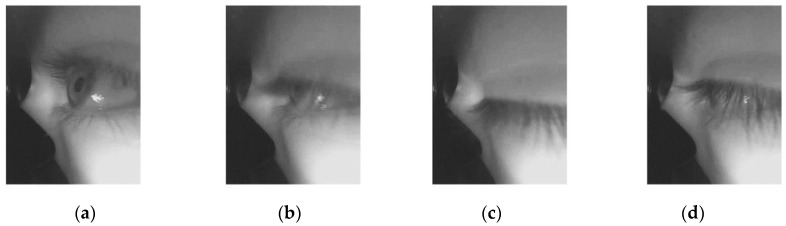
Eyeblink phases: (**a**) An open eye; (**b**) Closing phase; (**c**) Pause phase; (**d**) Reopening phase.

An eyeblink is defined as “a temporary closure of both eyes, involving movements of the upper and lower eyelids” [10] (p. 111). They play an important role in the preservation of the eye’s ocular surface integrity. Previous studies have divided eyeblinks into three different phases: the closing phase, closed or pause phase and the reopening phase [11,12], as shown in Figure 1.

There are three different kinds of blinking that can occur: a spontaneous blink, which occurs unintentionally; a voluntary blink, which occurs consciously and lasts longer than spontaneous blinks; and a reflex blink, which occurs unconsciously due to external triggers, for instance, a puff of air in front of the eye, strong sound or light. Reflexive eyeblinks can last for a shorter duration compared to voluntary and spontaneous blinks [11,12,13]. The foremost were excluded from this study, because they are triggered at irregular intervals and are caused by an external stimulus. The blink rate is expressed as the mean rate of eyeblinks per minute. A healthy adult blinks roughly 10 to 20 blinks min^−1^ [13,14]; each eyeblink can last between 75 and 400 ms [13,15,16]. This range includes spontaneous and reflexive eyeblink classes. On the other hand, the volunteer eyeblinks have no defined duration. A few studies have investigated the development of blinking activity according to age [17,18,19], finding an increasing in eyeblink rate in children up to adolescence [17], while this becomes stabilised in adulthood [14,18]. In other studies, the eyeblink rate in elderly subjects was found to show no significant difference when compared to younger adult subjects [14,18,19,20]. During work conducted by Bologna–Agostino et al. [11], the kinematic variables of voluntary and spontaneous eyeblinks for healthy subjects were observed. Table 1 shows some of these variables, which are closing, pause and reopening durations for voluntary and spontaneous eyeblinks. In Section 2, several eyeblink detection methodologies and other related studies are briefly reviewed.

## 2. Related Studies

Many approaches have been developed to detect eyeblinks for different applications in the domain of human–computer interaction and they can be divided into three major categories based on the data acquisition method. Approaches in the first category utilise physiological measures [21,22], for instance, employing electro-encephalography (EEG), or electro-oculogram (EOG) [23,24]. The approaches in the second category utilise an optical proximity sensor to detect eyeblinks. For instance, a study conducted by Ishimaru et al. [9] focused on combining head motion patterns and eyeblink frequency. This information can be used to distinguish different types of high-level activities like reading, watching TV, talking and solving a mathematical problem. In this study, eyeblink frequency was estimated using the Google glass proximity sensor, which detects eyelid closure. As mentioned earlier, the approaches in the first and second categories are beyond the scope of this paper.

The approaches in the third category utilise image sensors to monitor the region of interest (the eye) to identify eyeblinks. Furthermore, methods are deployed to classify eye state (open or closed). For instance, Al-gawwam [25] utilised an image sensor (camera) to track the facial landmarks’ positions to localise the eyes and eyelid contours. Later, the vertical distance between the upper and the lower eyelids was estimated after signal prefiltering using a Savitzky–Golay filter to improve signal-to-noise ratio (SNR). A finite state machine (FSM) was used to distinguish between false and true blink detection based on blink duration. Another method was proposed by Fogelton [6], which was based on motion vector analysis. The proposed approach generates an eyeblink signal based on the vertical component of the calculated motion vector at the eye region. A finite state machine was used for decision making to identify eyeblinks. The performance of the automatic eyeblink detection method was good (95.8% and 91.7% for precision and recall, respectively). However, the author did not mention how the values of eyeblink detection threshold were estimated, and how the proposed approach selects the proper detection threshold from these values. Additionally, the processing time for a single frame was around 20 milliseconds, which makes this method inadequate for our application. Another approach was proposed by Królak [7], which detects voluntary eyeblinks based on time constraints. The method utilises a Haar-like feature classifier to localise the face and the eye in the captured image. In the next step, an eyeblink signal is generated by using cross-correlation with an open eye template image over time. By comparing the signal with a predefined detection threshold, it is possible to detect eyeblinks. However, the processing time required for each frame is quite long (156 ms for 320 × 240 pixels frame size, for only face and eye localisation). Moreover, the author did not show how the eyeblink detection threshold value had been calculated.

Likewise, during a study conducted by Ivleva [26], an eyeblink detector was implemented using a light-dependent resistor (LDR) and green light-emitting diode (LED), which detects eyelid closure, thus indicating eyeblinks. However, the proposed method suffers from a major drawback, which is the low accuracy and high rate of false positive detections due to using a Fixed LDR for eyeblink detection. Furthermore, the paper did not provide any evaluation of the performance of the eyeblink detection method.

A considerable amount of research has been carried out related to head-mounted eye tracking. For instance, in a study conducted by Fuhl et al. [27], a new method for identifying the eyelid and estimating the eye aperture was proposed. The eye aperture was measure by approximating the lower and the upper eyelids using oriented edges. Furthermore, during the study, a new dataset was collected to validate the proposed approach, which has been published online for validation of similar methods in future studies. In another study conducted by Fuhl at el. [28] an eyeblink detection method for head-mounted eye-trackers was put forward, which is brightness and motion-based. The proposed approach does not utilise pupil or eye localisation or edge detection. It is based on the following assumptions: the pupil is dark, and it gets fully or partially occluded by the eyelid during eyeblinks. Consequently, the brightness in the captured frames will be changed. Based on these assumptions, the algorithm subtracts two sequential frames (both framed are blurred for noise reduction) to generate a features image and uses percentile values to measure its brightness. Afterwards, a sequence of successive frames determined by sliding window width is used to extract the features from the frames sequence. The proposed algorithm utilises the Random Forest Classifier [29] for identifying eyeblinks. The approach has been validated using a challenging dataset based on a real case scenario. The author reported that its shows high detection rate of 96.3795% using the 50th percentile and a window size of 11 frames. Moreover, the algorithm is not computationally expensive. However, the proposed algorithm identifies the eyeblink after the complete blink event has already occurred. In other words, the eyeblink is identified after the reopening phase has ended. Furthermore, the algorithms in both studies used a frontal eye monitoring setup, which makes the dataset collected inappropriate for our VR application.

Most of the reviewed methods have involved employing frontal eyeblink monitoring setups using a head-mounted eye-tracker, such that the camera positioned directly above the eye do not utilise an eye localisation procedure to identify the eye in the captured frame. However, other methods monitor the whole face and require an eye localisation approach in order to localise the eye region. Nevetheless, these techniques cannot be adopted in our approach, because the VR headset covers both of the participant’s eyes. Due to the limited space inside the VR headset, it is not possible to use head-mounted eye trackers combined with the VR headset. Hence, we decided to mount the monitoring camera inside the headset, but this can cause display occlusion while using the frontal eye monitoring setup. So, a non-frontal monitoring setup was deemed more appropriate for our approach. As a result, the available datasets in the community were non-applicable with our method. Additionally, eyeblink detection occurs after the reopening phase has finished, which is too late for the requirement of our application. The next section discusses our proposed method and the challenges of the design in detail.

## 3. Design Problem Statement and Proposed Method

In VR, the participant wears a VR headset (head-mounted display, HMD) to perceive a VR experience. The design challenges of detecting eyeblinks when someone is wearing a VR headset are different when compared with the conventional methods due to eye occlusion in the former. Furthermore, in our approach, a non-frontal eye monitoring setup was used. Accordingly, frontal eyeblink detection approaches cannot be used in our implementation. In this study, the main objective behind implementing an eyeblink recognition system is to reposition and reorient the participant imperceptibly in the IVE during eyeblinks. Hence, eyeblink detection should be significantly accurate and as fast as possible so that enough time is available to change the participant’s position and orientation. However, the available eyeblink identification approaches detect eyeblinks after identifying the reopening phase, which does not give enough time to reposition or rotate the participant during eyelid closure. Consequently, the available approaches were just a starting point for developing and validating our proposed approach. Additionally, we designed the solution such that it does not require preparation or calibration each time it is started. Below is a list of the design problem statements for the proposed approach:

- No calibration is required;
- The sensor should be mounted inside the VR headset;
- No markers and electrodes are attached to the participant’s face;
- Eyeblinks are identified during the closing phase;
- Processing time for a single frame is <13 milliseconds (≈73 frames per second);
- A single camera should be used to capture the eye movement inside the VR headset as this will have a lower cost and require fewer computational resources.

Our proposed method utilises a high-speed USB camera equipped with an OV4689 image sensor [30] to monitor the eye inside the VR headset. The captured real-time video stream has been processed based on the following assumptions:

- In the captured video stream, there are no moving objects except for the eyelid and the ocular ball;
- Eyelid movements cause the predominant amount of motion in the video stream;
- The motion directions caused by eyelid closing and reopening are parallel to the vertical axis (*y*) in the captured video stream images;
- During the pause phase of the eyeblink, the eye is fully closed.

Based on these assumptions, the proposed method calculates a motion vector between two successive frames to identify eyelid closure, thus indicating the closing phase. Afterwards, full eyelid closure is verified to identify the pause phase in order to indicate an eyeblink. Before describing the proposed methodology in detail, we explain the challenges of recognising eyeblinks in real time utilising an image sensor inside a VR headset.

### 3.1. Challenges of Recognising Eyeblinks for a VR Headset

We had to address several problems regarding identifying eyeblinks for VR headset, which can be summarised as follows.

#### 3.1.1. Lighting Conditions

To obtain a clear view of the eye inside the headset, effective illumination is required and the only available visible light source inside a VR headset is the display itself. However, this is not a reliable lighting source, because the amount of luminance intensity varies according to the scene currently displayed on the headset screen. Variable illumination intensity causes the dense optical flow algorithm of Gunnar Farneback [31] to estimate a false motion vector, because it utilises the change in pixel intensity to calculate the dense optical flow between two successive frames [31]. Hence, an external light source is required to illuminate the eye inside the VR headset. However, using visible light for illumination will raise other problems:

- Visible light will distract the participant and might affect their natural eyeblink kinematic variables;
- The amount of illumination intensity will vary due to changes in the brightness intensity of the display inside the headset, which, as mentioned above, will cause distortion in the estimation motion vector between two successive frames in the video stream.

We came up with a solution of using near-infrared LEDs (λ 850 nm) [32] as a lighting source inside the VR headset to illuminate the participant’s eyes. We also used an IR pass filter in front of the image sensor to reject the visible light caused by the VR headset’s display.

#### 3.1.2. Eyeblink Kinematics

**Figure 2:**
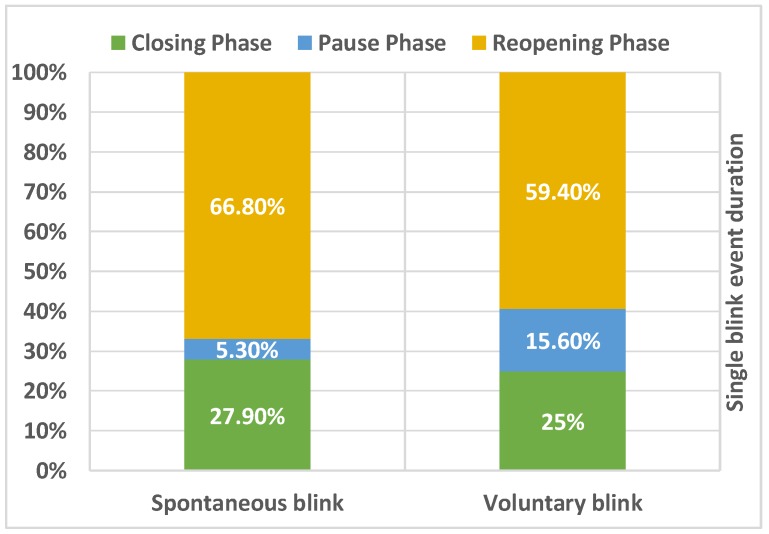
The proportion of each phase with respect to a single blink event, for both spontaneous and voluntary eyeblinks. The calculated values are based on a dataset for healthy controls collected during a study conducted by [11].

For real-time eyeblink detection, a closer look should be taken at the duration of the blink’s kinematic variables. As aforementioned, a complete eyeblink for healthy subjects can last between 200 and 400 milliseconds. We performed the following calculations on the data in Table 1 to estimate the approximate percentage duration of each eyeblink phase with respect to the total duration of the eyeblink. These calculations gave a better overview of the duration of each different phase during a single eyeblink event and helped to determine the appropriate capture frame rate (FPS) required from the camera. Hence, Table 1 can be extended, as shown in Table 2 and its visualisation in Figure 2, where:

(1) $$\begin{array}{l} Total\;blink\;duration\\ \;=\sum (Closing\;phase+Pause\;phase\\ \;+\;Reopening\;phase)\;durations\;in\;ms \end{array}$$

(2) $$Percentage\;of\;the\;phase=\frac{Phase\;duration}{Total\;blink\;duration}\times 100\%$$

Regarding the tolerance value in the total blink duration, we have simply considered the highest tolerance value, which are ±15.9 and ±13.8 ms for voluntary and spontaneous eyeblinks, respectively.

Figure 2 shows the approximate percentage duration of each eyeblink phase (regardless of the difference between the duration of each blink category) with respect to a complete single blink for both spontaneous and voluntary blinks. As can be seen, the closing and pause phases together can take around 33% to 41% of the complete eyeblink duration, which is approximately 85 and 116 milliseconds (based on the data in Table 2) for each spontaneous and voluntary eyeblink, respectively. Additionally, spontaneous blinks had a shorter pause phase compared to other focal blink types (please refer to Table 2 in [11]). Therefore, in order to calculate the worst-case scenario, we considered the spontaneous eyeblink in the following calculations.

As shown in Figure 2, the pause phase comprises about 5.3% of the total blink duration, which is 13.7 ± 3.3 milliseconds. If a camera with a 30 FPS capture rate has been utilised to track eyeblinks, it is probable that at some point it will miss the pause phase event during spontaneous blinks, because ΔT is greater than the pause phase duration:

(3) $$\Delta T=\frac{1}{FPS}\to \frac{1}{30}\approx 33\;\mathrm{ms}\;>\mathrm{pause}\;\mathrm{phase}\;\mathrm{duration}\;\left(13.7\pm 3.3\;\mathrm{ms}\right)$$

This leads to inaccurate eyeblink tracking. It is possible to estimate approximately the minimum frame per second required ($FPS_{required}$) to capture eyeblinks more reliably based on the parameters from Table 2 using the following equation:

(4) $$FPS_{required}=\frac{1}{\Delta T_{minimum}}\to \frac{1}{0.0137}\approx 73\;FPS$$

where, Δ*T_minimum_* is the minimum duration between two successive captured frames, which is assumed to be equal to or less than the pause phase duration. In the next section, the system design and the proposed algorithm are discussed in more detail.

### 3.2. Hardware System Design

**Figure 3:**
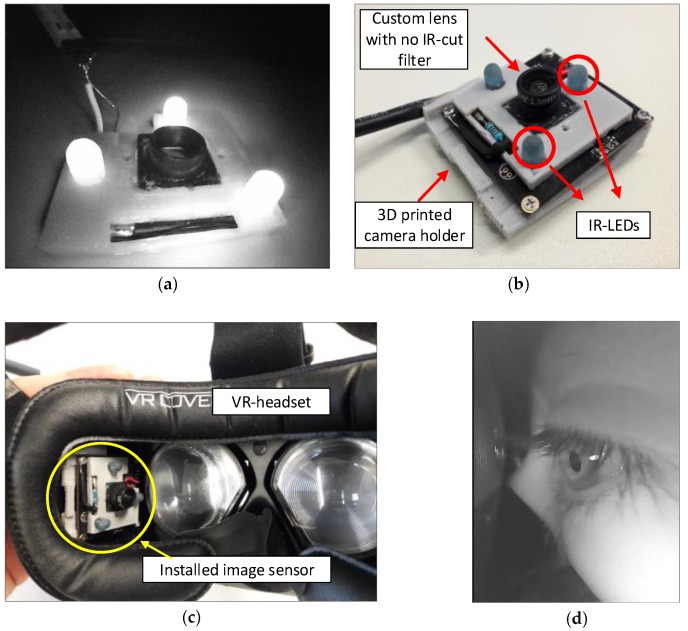
The modification on the monitoring camera: (**a**) Infrared spectrum image for lens mount M8 and IR-LEDs for internal illumination in action; (**b**) Adding of the custom lens with no IR-cut filter, infrared LEDs for eye illumination and custom 3D printed camera holder; (**c**) Installing the camera inside the VR-headset (HTC-Vive); and (**d**) Image sample captured by the camera inside the VR headset.

Near-infrared LEDs were used to illuminate the eye inside the VR headset, whilst a high-speed USB camera was utilised to monitor the eye. However, behind the camera lens, there was an IR-cut filter, which rejects infrared-light and thus, some modifications needed to be made. These modifications comprised the replacement of the default camera lens (100° field of view (FOV) with an IR-cut filter) with a 145° FOV no IR-cut filter, such that the sensor can be exposed to near IR light. Finally, the camera was mounted on a customised 3D printed holder so that it could be fixed securely inside the VR headset. Figure 3 shows the modifications to the USB camera.

Regarding the implementation of the illuminance circuit of the eyeblink detector, we used three IR-LEDs TSHG6400 from Vishay [32] to illuminate participant’s eyes inside the VR headset, with Table 3 showing the LED’s summarised specifications. More technical specifications can be found on the official website of Vishay.

**Figure 4:**
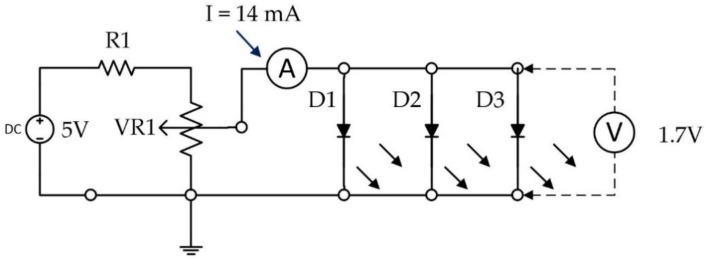
Schematic diagram of the illumination circuit.

The LEDs have ±22 angle of half intensity, which makes it inefficient for illuminating near objects. Hence, the LEDs had to be diffused by sanding them with sand paper to scatter the light to achieve better illumination. The illumination intensity of the LED is powerful and, theoretically, it is possible to use one instead of three. However, due to the narrow radiation angle, it was difficult to illuminate evenly the eye without operating the LED at the maximum radiant power. This is not a desirable working point for the LED, especially as it is very close to the eye. Hence, three LEDs were used to illuminate the eye evenly and were kept running at a low operating point to avoid over-illuminating the eye. The operation point of these LEDs was 1.7 V and approximately 5 mA for each. Figure 4 shows a schematic diagram of the illumination circuit.

### 3.3. Detection Algorithm

**Figure 5:**
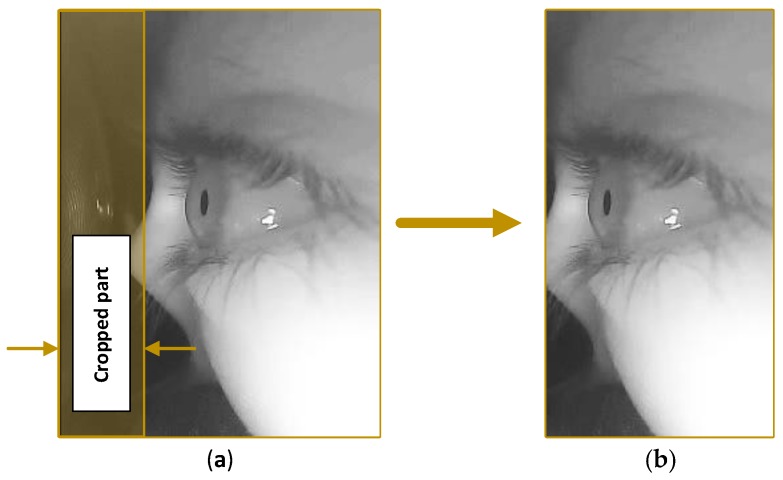
The captured frame: (**a**) Captured frame before the crop, with frame resolution 240 × 320 pixels; (**b**) Captured frame after the crop, with frame resolution 170 × 320 pixels.

Generally, the detection algorithm comprises three different phases: *initial blink detection*, *blink verification* and *reopening phase detection.* Two successive frames (*f_n_*_−1_, *f_in_*) were captured; the resolution of each captured frame is 240 × 320 pixels for the width and height, respectively. Because different people have different facial structure, eye shape and size, the frame orientation is in portrait layout to cover as much of the facial area as possible and to obtain a clear view of the eye. During dataset collection, we observed that the first 70 pixels do not hold any useful information, because they show the inner side of the VR headset. So, we decide to crop the first 70 pixels of the frame to improve the performance, thus reducing the frame size to 170 × 320 pixels for the width and height, respectively, as shown in Figure 5.

#### 3.3.1. Initial Eyeblink Detection Phase

Based on the assumption made earlier (there are no moving objects in the captured frames except the eyelid and the ocular ball), during the closing phase of the blink, the upper eyelid will move down in a rapid motion to cover the ocular ball. However, the lower eyelid is almost stationary and hence, the main motion direction between the captured frames is pointing downwards during the closing phase. During the initial blink detection phase, the detection algorithm utilised the Farneback algorithm [31] to estimate the dense motion between the successive frames and thus, estimate the overall motion vector to detect the closing phase.

**Figure 6:**
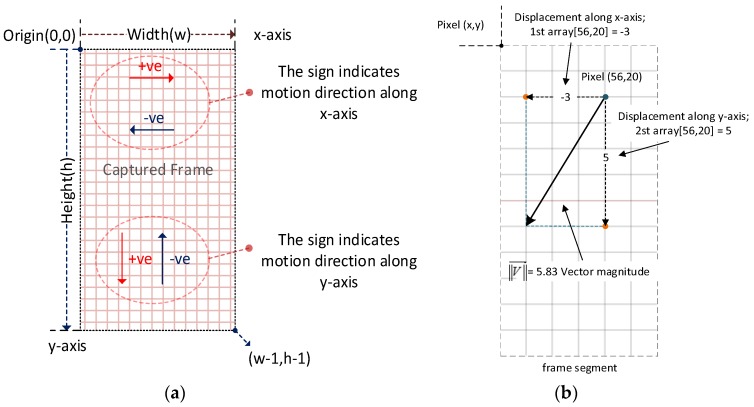
Image coordinates and motion vector calculation: (**a**) Image 2D coordinates, with the origin being located at the top left corner, the *x*-axis is pointing to the right and the *y*-axis is pointing downwards. The sign of the magnitude of the vector component (*x* and *y* values) indicates the direction of motion; (**b**) Frame segment and calculating the motion vector for a single pixel.

The output of the Farneback algorithm comprises two 2D arrays, both of which have the same size as the input frames. Each element in the array refers to a pixel on the captured frame, and it holds the distance of how far that pixel has moved with respect to the previous frame in pixel units. Each element in the first array holds the travelled distance along the x-axis for a particular pixel, whereas the elements held by the second array represent the travelled distance along the *y*-axis for that particular pixel. Based on pixel location in the previous frame and both arrays, as calculated by the Farneback algorithm, motion vectors can be estimated. These vectors indicate motion direction between two successive frames and Figure 6 explains how these vectors are estimated.

Figure 6a illustrates the 2D image coordinates, with the origin point being located at the top left corner of the frame. The *x*-axis is aiming to the right and the *y*-axis is aiming downwards. So, this is different to the conventional 2D Cartesian coordinate system, where the *y*-axis is aiming upwards. As mentioned before, each value held by the two arrays, calculated by the Farneback algorithm, expresses how far each pixel has moved along the *x*- or the *y*-axis. If the value is equal to zero, this indicates that no motion occurred along the specified axis, whereas, if it is less than zero (negative), this indicates motion in the decremental direction of the axes. In contrast, values of more than zero (positive) indicate motion in the incremental direction of the axes, as shown in Figure 6. Figure 6b shows a segment of the captured frame, how a single vector has been calculated from the start point (pixel location) and the displacement values for the both *x* and *y* axes.

**Figure 7:**
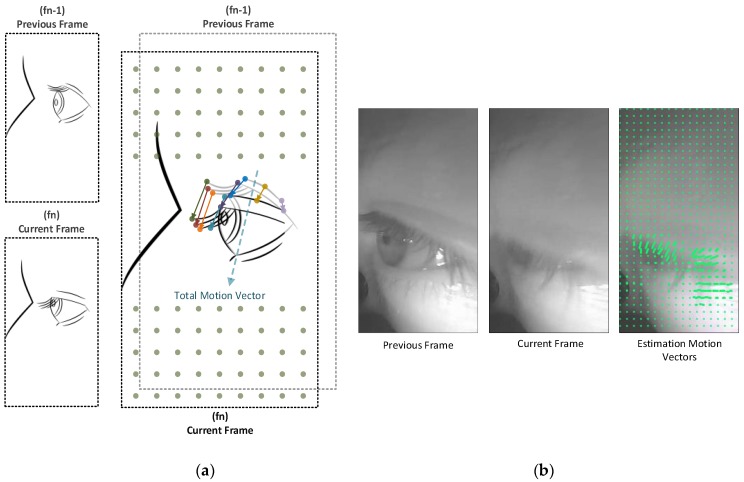
Illustration of an approximate estimation of a motion vector between two successive frames: (**a**) Two successively captured frames and how the motion vectors are estimated based on using the previous and current captured frames (*f_n_*_−1_, *f_n_*_,_ respectively), and how the sum of vectors represents the motion vector; (**b**) Real-case scenario, two captured frames and visualisation of the estimation motion vectors by the proposed algorithm.

Because the tracked object (the eye) takes up quite a large part of the captured frame, it is not necessary to use all the pixels in the image to estimate the motion vector. A set of points are distributed evenly on the previous captured image, which act as markers indicating the starting points for a set of vectors, as shown in Figure 7. The distance between each point to another is about “*d*” pixels, for each *x*- and *y*-axis, as shown in Figure 7. In our case, *d* = 10 and this procedure will reduce the processing time. The total count of points “*n*” is calculated as follows:

(5) $$n=\frac{w_{frame\;width}\times h_{frame\;height}}{\begin{array}{c} d_{pixels\;for\;X\;axis}\times d_{pixels\;for\;Y\;axis} \end{array}}\overset{yields}{\to }\;\frac{170\times 320}{10\times 10}=544$$

Figure 6 illustrates the process of estimating the overall motion vector from two successive frames by the Farneback algorithm. The summation of these vectors, indicating the overall motion vector ${\vec{MV}}_{total}$ between the last successive frames, is as illustrated in the following two equations:

(6) $${\vec{MV}}_{total}=\overset{n}{\underset{0}{\sum }}{\vec{\alpha }}_{n}$$

where:

(7) $${\vec{\alpha }}_{n}=\left[X_{w},\;Y_{h}\right]$$

*X_w_* and *Y_h_* represent the drift of a predefined pixel at $\left(w_{width},\;h_{height}\right)$ along the *x*- and *y*-axes, respectively, in pixel units on image coordinates. Both are estimated using the Farneback algorithm for calculating the optical flow, where ${\vec{\alpha }}_{n}$ is a vector describing the motion caused by the drift (*X_w_*, *Y_h_*).

As mentioned before, during the closing phase, the major downward motion is caused by the eyelid closure. However, the saccades phenomenon (which is rapid eye movement that occurs during gaze switching between two visual fixations) could also cause motion in various directions, depending on where the fixation points are before and after the gaze switching. Accordingly, saccades can cause false positive detection. To solve this issue, the following selection criteria were applied to the vectors used to calculate the total motion vector based on the magnitude and the direction of these vectors:

- Each vector with a magnitude less than two pixels will be discarded;
- Each vector with a negative y-component (pointing up) will be discarded;
- Each vector with a positive y-component and contributing to less than 50% of the total vector magnitude will be discarded.

After applying the selection criteria, the remaining vectors are added together to estimate the downward motion vector (DMV). If the magnitude of the downward motion vector $\left\|{\vec{MV}}_{down}\right\|$ is above a specific threshold, this probably indicates an eyelid closing and thus, an initial blink is registered.
**Method:** Estimating DMV (previous frame, current frame)
(a)Pass frames to the Farneback algorithm to estimate the overall motion vector (previous frame, current frame).(b)Apply the following selection criteria:
Subtract all vectors with a magnitude <2 pixels $\left(\|{\vec{\alpha }}_{n}\|<2\right)$;Subtract all vectors that have a Y component <0 (pointing upwards);Subtract all vectors that have a Y component >0 (pointing downward) that contributes <50% of their magnitude.


Equation (8) describes how the DMV ${\vec{MV}}_{down}$ is estimated.

(8) $${\vec{MV}}_{down}={\vec{MV}}_{total}-\overset{n}{\underset{0}{\sum }}{\vec{\alpha }}_{n}\;{|}_{\|{\vec{\alpha }}_{n}\|<2}-\overset{n}{\underset{0}{\sum }}{\vec{\alpha }}_{n}{|}_{Y<0}-\overset{n}{\underset{0}{\sum }}{\vec{\alpha }}_{n}{|}_{Y<\left(\|{\vec{\alpha }}_{n}\|\times 0.5\right)}$$

#### 3.3.2. Estimating the Dynamic Threshold for Eyelid Closure Detection

To achieve reliable eyelid closure detection based on DMV analysis, dynamic threshold estimation for eyelid closure is essential. The following algorithm describes how the dynamic detection threshold was calculated.

**Method:** Estimation of dynamic threshold for eyelid closure:
(a)if the $\left\|{\vec{MV}}_{down\left(i\right)}\right\|$ > 0;Add the $\left\|{\vec{MV}}_{down\left(i\right)}\right\|$ the current frame to the set X;(b)if the value of $\left\|{\vec{MV}}_{down\left(i\right)}\right\|$ <= 0, this indicates the end of the closing phase and the start of the pause phase;
○Y= maximum value in the set “X”;○Add Y to the thresholds set “T”;○New threshold value = finding the median value of the “T” set;

Else, repeat the first step (a).

- i represents the current frame number
- ${\vec{MV}}_{down\left(i\right)}$ represents the magnitude of the DMV for the current frame, $f_{i}$, with respect to the previous frame, $f_{i-1}$
- “*X*” is a set of values where each represents the magnitude of the DMV for *i*-th frame where the magnitude is >0:
  (9) $$X=\left\{\|{\vec{MV}}_{down\left(i\right)}\|\;:\;\|{\vec{MV}}_{down\left(i\right)}\|>0\right\}$$
- “*Y*” represents the maximum value in the “*X*” set, while *c* represents the count of values in “X”:
  (10) Y=max({f(x) : x=1,…,c }), Y∈X
- “Z” represents a set of $Y_{n}$ values that have been detected during the last *n*-th eyeblink:
  (11) Z={Y:Y=1,…,n}
- $T{}^{\prime }$ represents the median value of the “Z” set, while *c* represents the count of values in “Z”. The values in “*Z*” set are sorted in increasing order:
  (12) $$T{}^{\prime }=\{\begin{array}{c} Y_{\left(\frac{c+1}{2}\right)},\;c\;is\;odd\\ \;\frac{1}{2}\times \left(Y_{c/2}+Y_{1+c/2}\right),\;c\;is\;even \end{array}$$

In other words, the “Y” variable illustrates the maximum peak of the magnitude of the DMV during eyelid closure, while “Z” pertians to a set of maximum peaks of the last *n*-th eyeblink. Additionally, $T{}^{\prime }$ defines the new estimated eye closure detection threshold by calculating the median value of “Z”.

#### 3.3.3. Eye Reopening Detection

During the reopening phase of the eyeblink, the upper eyelid will move upward to reveal the ocular ball. Accordingly, estimating the upward motion vector ${\vec{MV}}_{upward}$ will indicate the reopening phase and the following equation shows how the upward motion vector was calculated:

(13) $${\vec{MV}}_{upward}={\vec{MV}}_{total}-\overset{n}{\underset{0}{\sum }}{\vec{\alpha }}_{n}\;{|}_{\|{\vec{\alpha }}_{n}\|<2}-\overset{n}{\underset{0}{\sum }}{\vec{\alpha }}_{n}{|}_{Y>0}$$

What follows is a description of the method utilised to estimate upward motion vector ${\vec{MV}}_{upward}$

**Method:** Estimating the upward motion vector (previous frame, current frame)
a)Pass frames to the Farneback algorithm to estimate the overall motion vector (previous frame, current frame);b)Apply the following selection criteria:
Subtract all vectors with magnitude <2 Pixels $\left(\|{\vec{\alpha }}_{n}\|<2\right)$;Subtract all vectors that have a Y component >0 (pointing downward).



#### 3.3.4. Localising the Eye “Region of Interest (ROI)” in the Captured Frame

**Figure 8:**
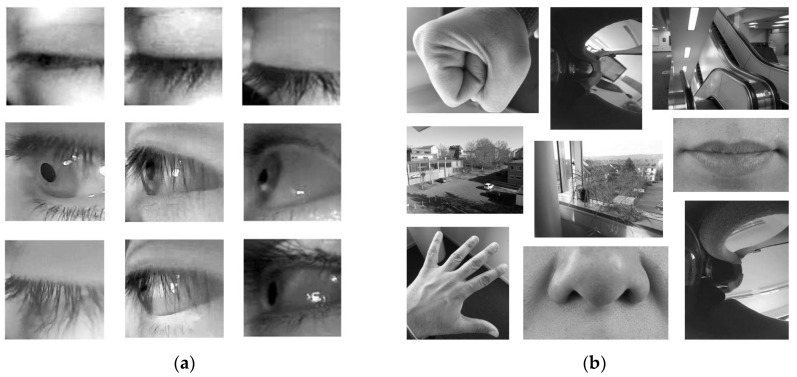
Sample images used to train the Haar cascade classifier (HCC): (**a**) Example of positive samples; (**b**) Example of negative samples.

Despite the fact that the image sensor was mounted directly above the eye, using a narrow FOV camera lens to avoid the eye localisation process was very difficult. This is because each participant has a different facial structure and hence, they might have a different interpupillary distance. Accordingly, they wear the VR headset differently, and during the VR session they might readjust the VR headset on their face for more comfort, which leads to misalignment between the eye and the camera. Consequently, eye localisation in our approach was inevitable. With our method, eye localisation was used to extract the eye feature from the captured frame to verify eyelid closure. The Haar cascade classifier (HCC) [33] was utilised to identify the ROI. The HCC was trained using 670 negative images and 168 positive images. The negative images contained different random parts of the body, while the positive ones had side views of the left eye region from several participants during different eye states (eye open, closed and partially closed). Figure 8 shows samples of the positive and negative images used in the HCC training.

The training was carried out using an open source application “Cascade Trainer GUI” [34]. This application was used to train and test cascade classifier models. After several trials of tuning different training parameters (such as window size, number of stages and minimal hit rate), the training session took around six hours to complete. The hit rate of the HCC class of the eye localisation method was around 0.998 and 0.003 for the mean and standard deviation, respectively. Table 4 shows statistical information about the eye localisation method hit rate. It should be noted that the dataset used for validation was not used during the training session. For more information about the Cascade Trainer GUI application please refer to [34]. The hit rate was calculated based on Equation (14).

(14) $$Hit\;rate=\frac{frame\;Count\;were\;ROI\;was\;detected}{Total\;frame\;Count}$$

#### 3.3.5. Verifying Eyelid Closure Using Cross-Correlation with a Template Image

**Figure 9:**
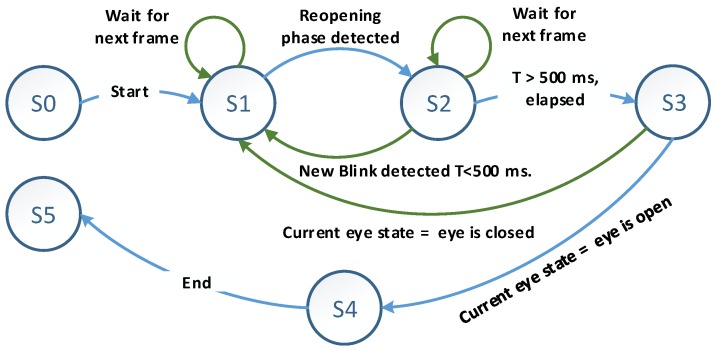
The finite state machine for open eye template image automatic selection.

During the next phase (blink verification), verification of eyelid closure takes place. After possible eyelid closure is detected, the localised ROI for the current frame, *f_n_*, is checked with a template image, *t* (an open eye). A normalised cross-correlation coefficient, *Q*, is calculated based on Equation (15) to check the similarity between the current ROI and the eye open template image to verify eyelid closure.

(15) $$Q\left(x{}^{\prime },y{}^{\prime }\right)=\frac{\sum_{x{}^{\prime }y{}^{\prime }}\left[f\left(x{}^{\prime },y{}^{\prime }\right)\times t\left(x,y\right)\right]}{\sqrt{\sum_{x{}^{\prime }y{}^{\prime }}f{\left(x{}^{\prime },y{}^{\prime }\right)}^{2}\times \sum_{x{}^{\prime }y{}^{\prime }}t{\left(x,y\right)}^{2}}}$$

where, −1≤Q≤1 and represents the normalised cross-correlation coefficient, *f* is the current ROI (which is the eye) and *t* is the open eye template image. If the value of the cross-correlation coefficient is near 1, this indicates high similarity between the test sample and the template images. Whenever eyelid closure is verified, an eyeblink will be registered. Otherwise, a new frame will be captured, and a new detection cycle will begin. In our proposed method, we used an automatic approach to select a template image for the open eye. Figure 9 illustrates the finite state machine (FSM) for template selection.

Figure 9 shows the FSM for automatic template selection used in our proposed approach, with there being six different states. “*S0*” and “*S5*” represent the initial and end state, respectively, while “*S1*” represents waiting until the reopening phase is being detected. “*S2*” represents time constrain, which is waiting for 500 milliseconds to elapse, a condition that will ensure the ending of all eyeblinks phases and enough time in case of double eyeblink occurrence. “*S3*” pertains to checking of the eye state (open/closed) and if it is “*eye closed*”, the current state will be set to “*S1*”. Otherwise, the current ROI will be considered as a template image for an open eye.

#### 3.3.6. Overall Structure of the Proposed Detection Algorithm

**Figure 10:**
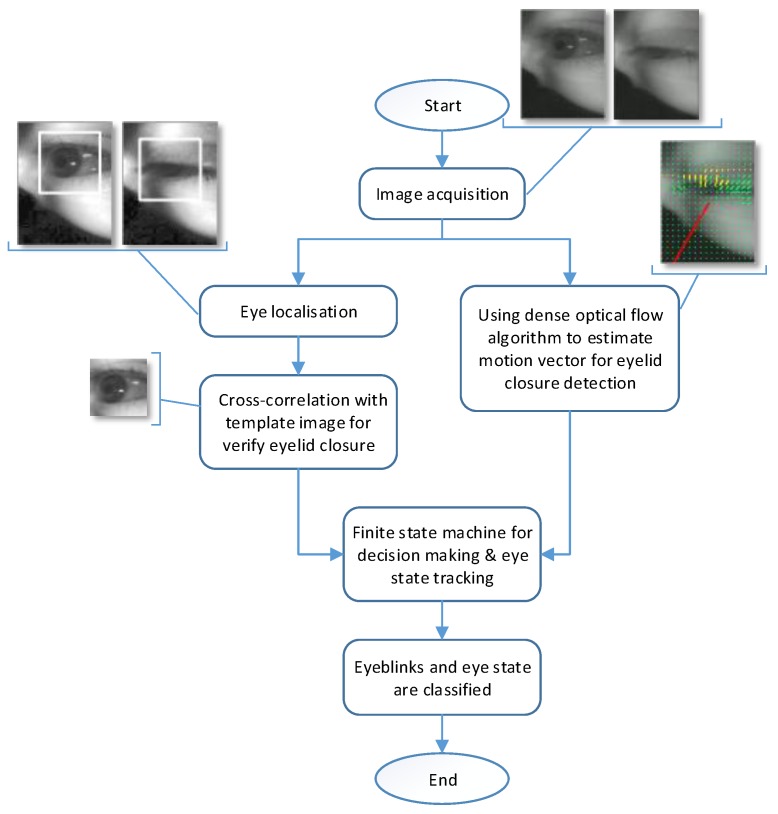
The structure for our eyeblink detection algorithm.

Figure 10 shows the structure of the eyeblink detection and eye state classification of the proposed method. Multithreading programming was utilised to reduce the time required for processing each frame. As shown in Figure 10, estimating the motion vector was achieved on a separated thread. Simultaneously, eye localisation and eyelid closure verification were performed on another thread. Please refer to Appendix A for more details about the proposed method in action.

Afterwards, a finite state machine was used for decision making to detect eyeblinks and classifying eye state based on the information processed by motion vector estimation, eye localisation and eyelid closure verification.

## 4. Finite State Machine for Eyeblink Detection and Classification

**Figure 11:**
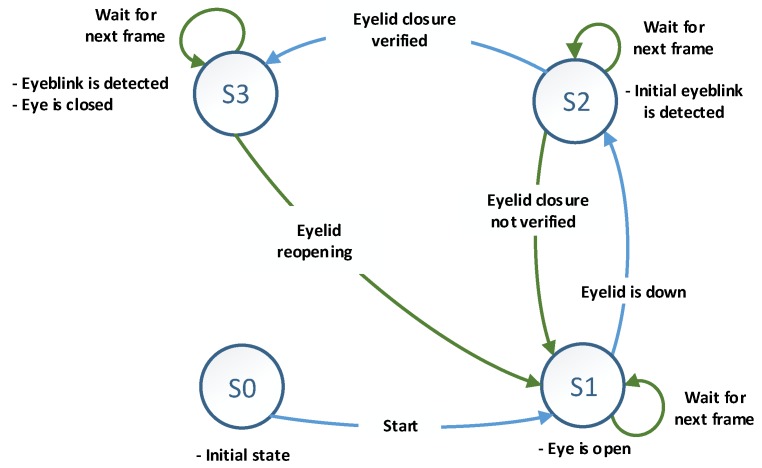
Finite state machine for identifying eyeblinks and tracking the eye state.

A finite state machine was defined for decision making to identify eyeblinks and classify the eye’s current state, as shown in Figure 11.

**Figure 12:**
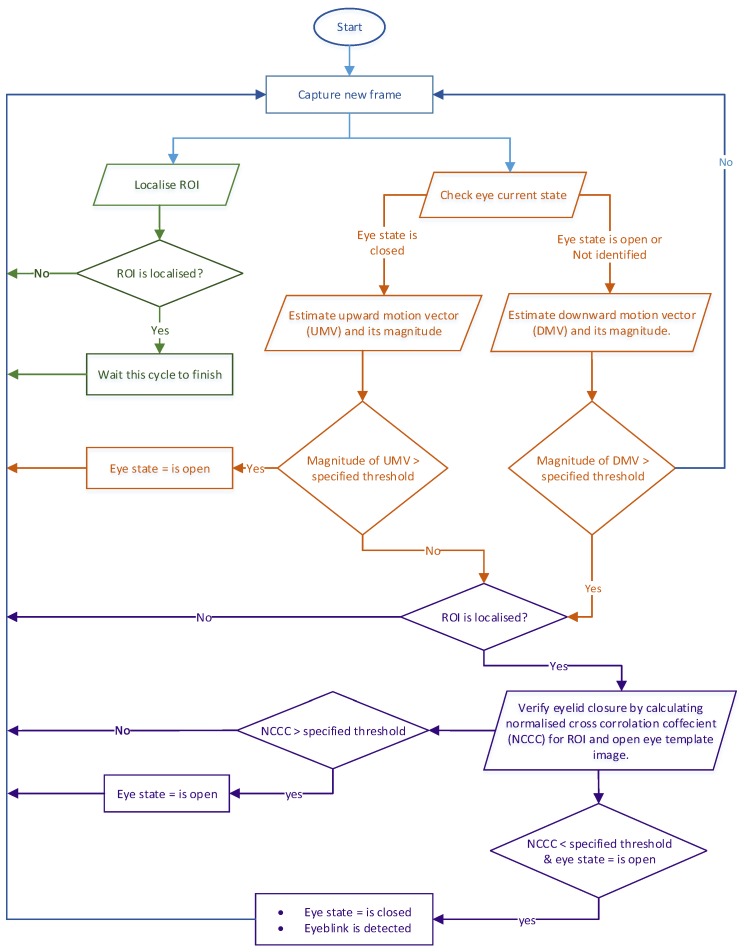
Eyeblink decision making logic diagram.

The FSM utilises the extracted information from motion vector analysis, the Haar cascade classifier for eye localisation, and cross-correlation between the ROI and open eye template image to identify an eyeblink and classify the eye state. As shown in Figure 11, the FSM consists of four different states: “*S0*” presents the initial state, while “*S1*” indicates the eye is in the open state and waiting for eyelid closure. “*S2*” pertains to initial eyeblink detection and eyelid closure verification. If eyelid closure is verified, “*S2*” will transit to “*S3*”, which indicates a valid eyeblink, and the eye state will be classified as closed. During “*S3*”, if eyelid reopening is detected, the current state will transition to “*S1*”, thus indicating that the eye has reopened, and a new detection cycle will begin. Figure 12 shows the logic diagram of the eyeblink detecting decision making process.

Figure 12 shows that there are two branches where the data could flow after the captured frame arrives, which are (1) localising the ROI and checking the eye current state, or (2) calculating the dense optical flow to estimate the motion vector. These branches are running on separated threads to reduce the processing time required for each frame. Based on the current eye state, estimating motion vector will be carried out and compared with the specified threshold. If eye state is open or not identified, the DMV will be calculated to check for eyeblink occurrence. When the magnitude of the DMV is above the specified threshold, an initial eyeblink will be registered. For the next stage, the eyelid closure will be verified with an open eye template image.

The normalised cross-correlation-coefficient will be calculated and if its value is under the specified threshold a blink will be registered, with the eye state being classified as closed. Otherwise, a new detection cycle will begin. When the eye state is closed, the reinitialisation will be triggered by estimating the UMV to calculate its magnitude. If the magnitude of the motion vector is above the specified threshold, the reopening phase will be detected. Accordingly, the eye state will be classified as open and a new detection cycle will begin. On the other hand, if the magnitude of the UMV is below this threshold, a secondary reinitialisation sub-routine will check the current ROI with an open eye template to detect the reopening phase.

## 5. Dataset for Training and Validation

One of the challenges that we faced due to using a non-frontal eye monitoring setup, was finding the proper dataset to train the classifier for localising the eye feature in the captured image. The available datasets in the community employed a frontal eye monitoring setup. Moreover, most of these datasets had different lighting conditions compared to the eye monitoring conditions inside a VR headset. Unfortunately, there were no datasets available for the non-frontal eye monitoring setup of a VR headset. Therefore, we collected our own dataset. A high-speed USB camera (OV4689 image sensor on board) was used with a capture frame rate between 100 and 110 fps, and the samples were recorded with an mpg2 file format with frame resolution 240 × 320 pixels for the width and height, respectively. However, this resolution was reduced further during the pre-processing to reduce the processing time required per frame. The dataset was collected from 12 different participants: two females and 10 males. The mean of their ages was 31.33 years, with standard deviation equal to 3.24. There is one video file for each participant, and they vary in duration. Table 5 shows the statistical information for the collected samples.

The dataset comprises video-recorded samples for monitoring a participant’s eye inside the VR headset from several participants. The video stream in the dataset samples was rotated 90 degrees counterclockwise to compensate for the physical camera rotation inside the VR headset. The reason for mounting the camera 90 degrees counter-clockwise was to simplify the hardware implementation, using easier cable management inside the VR headset. Theoretically, camera orientation should not influence the algorithm’s operation. If we had taken the camera rotation into account from the beginning, we would have used the right and the left direction motion vectors for detecting eyelid closure and eyelid reopening, instead of downward and upward motion vectors, respectively. However, we chose to rotate the captured video stream to compensate for the camera’s rotation for a better understanding of the captured frames from a human perspective. During the sample collection, the participants were asked to explore their surrounding virtual environment (VE) and engage in interaction with virtual objects in the VE. We found this step necessary for distracting them from the fact that their eyes were being recorded, thereby reducing the number of possible voluntary eyeblinks.

### 5.1. Dataset Annotation

Dataset annotation can be defined as a high level of information added to the raw dataset as labels or tags that describe a specific role or class in the dataset. Additionally, it helps to train and validate machine-learning algorithms. The annotations file consists of an excel file with several separate sheets. Each sheet relates to a raw sample video file and the whole file represents the log of the method’s output. The following data were recorded automatically: frame number, magnitude of the downward and upward motion vectors, current eye state, detection of an eyeblink and ROI in the current frame detection. Two other columns, ground truth and the category of the detected eyeblink (true positive TP, false positive FP or false negative FN), were added to the sheet manually. Each video sample was reviewed and observed frame by frame to identify the actual eyeblinks that occurred during the recording. These annotations were used to compare the detected eyeblinks with the real ones, to evaluate the performance of the proposed method. The collected raw samples and their annotations file have been published on Mendeley database for datasets [35] under the following *DOI* “DOI: 10.17632/9yzdnng594.1”. We decided to publish them, because we believe they might be useful for future similar studies.

## 6. Performance Evaluation

Our evaluation procedure is based on receiver operation characteristics (ROC) analysis that has been reviewed by Fawcett [36]. It is a method utilised to analyse and visualise classifier performance. In this study, we used this approach to validate and evaluate the performance of our method. We now explain some terminology relating to eyeblink performance evaluation. Ground truth (GT) refers to the actual eyeblinks that exist in the eyeblink signal and true positive (TP) to the detected eyeblinks that intersect (exist) with the ground truth. In contrast, false negative (FN) pertains to an eyeblink that exists in the GT, which is not detected by the algorithm, while false positive (FP) refers to an identified eyeblink that does not intersect (does not exist) with the GT. Detected blinks (DBs) represent the total count of the overall detected eyeblinks in the tested samples.

Recall describes the sensitivity or the hit rate of the proposed method, and it can be expressed by Equation (16). Precision pertains to how many selected items are relevant, representing the performance of the method, and it is expressed by Equation (17). Additionally, false discovery rate (FDR) is expressed by Equation (18) and, finally, the overall accuracy can be defined by Equation (19).

(16) $$Recall=\frac{TP_{count}}{TP_{count}+FN_{count}};$$

(17) $$Precision=\frac{TP_{count}}{TP_{count}+FP_{count}};$$

(18) $$FDR=\frac{FP}{FP_{count}+TP_{count}};$$

(19) $$Accuracy=\frac{TP_{Count}}{TP_{Count}+FP_{Count}+FN_{Count}}$$

Based on Equations (16)–(19), Table 6 shows the performance of the proposed approach.

## 7. Conclusions and Directions for Future Work

In this study, we proposed a method for detecting eyeblinks and classifying eye state in real time for VR headsets. Furthermore, a prototype for an eyeblink detector was implemented utilising a high-speed image sensor. The proposed method can detect eyeblinks during the closing and the pause phase of the blink. The processing time required for each frame takes 9 to 11 ms and the estimation of the motion vector takes approximately 80% of this time. Our proposed method delivers 83.9% accuracy, 91.8% for precision and 90.40% for the recall. During the study, we observed that the main reason for detecting FN blinks in the proposed method was the micro blinks that can occur during the saccades (visual gaze switching between two fixation points). Most of the time, these micro blinks are below the detection threshold. Similarly, saccades that occur along the vertical axis of the field of view can cause FP eyeblink and selecting the appropriate template for eyelid closure verification plays a big role in reducing FP eyeblinks. As a direction for future work, improving the method for automatically selecting the appropriate template image for eyelid closure verification will have a high impact on the overall performance of the algorithm.

Furthermore, we have noticed that selecting the optimal position of the camera inside the VR headset can contribute to reducing the count of false positive detections. The optimal position is using a frontal eye monitoring setup. However, in the proposed approach, a non-frontal eye monitoring setup has been used for several reasons: first, a frontal eye monitoring setup is difficult to use, because the camera might occlude the screen of the VR-headset, which will reduce the field of view. Second, the available space inside the VR headset (HTC Vive VR headset) is limited; the minimum size of the affordable high-speed USB camera (120 fps) we could access at the time of conducting this study was 28 × 28 mm for the camera board. A smaller camera size will help to monitor the eye from a better angle, thereby reducing FP detection. As mentioned before, 80% of the processing time of each frame was used for estimating the motion vector in the captured frame. We believe that the processing time can be reduced further to speed-up the eyeblink detection method, which can be achieved by using another algorithm faster than the Farneback algorithm for calculating the dense optical flow to estimate the motion vector between two successive frames. Regarding the collected dataset, it is now available online with its annotation file (please refer to Section 5.1 regarding accessing the dataset). We hope it will be useful for future studies.

## Figures and Tables

**Table 1:** Durations of three kinematic variables for two types of blinking (voluntary and spontaneous) for healthy subjects in milliseconds [11].

Kinematic Variable	Voluntary Blinking	Spontaneous Blinking
Closing duration	70.8 ± 4.3	71.5 ± 4.7
Pause duration	44.2 ± 9.6	13.7 ± 3.3
Reopening duration	168.5 ± 15.9	171.5 ± 13.8

**Table 2:** Some of the blink kinematic variables: duration of each phase for voluntary and spontaneous blinks. The columns represent the percentage of each phase duration of the whole blink duration, which was roughly calculated for a better understanding of the different durations. The total blink duration was estimated by the summation of that of each phase (closing, pause and opening).

Kinematic Variable	Voluntary Blink	Percentage	Spontaneous Blink	Percentage
**Closing duration**	70.8 ± 4.3	~25%	71.5 ± 4.7	~27.9%
**Pause duration**	44.2 ± 9.6	~15.6%	13.7 ± 3.3	~5.3%
**Reopening duration**	168.5 ± 15.9	~59.4%	171.5 ± 13.8	~66.8%
**Total blink duration**	~283.5 ± 15.9	---	~256.7 ± 13.8	---

**Table 3:** Summarised technical specifications of the IR-LED.

Component	I_ө_ (mW/sr)	Φ (deg)	λ_p_ (nm)	t_r_ (ns)
TSHG6400	70	±22	850	20

**Table 4:** Frames count in the tested video samples and the eye localisation method hit rate.

Sample File Name	Total Frame Count	Localised ROI Frame Count	Hit Rate
S1	31,071	31,071	1
S2	32,230	32,230	1
S3	41,388	40,972	0.989948777
S4	8720	8719	0.999885321
S5	34,355	34,354	0.999970892
S6	20,460	20,459	0.999951124
S7	32,074	32,055	0.99940762
S8	11,278	11,277	0.999911332
S9	57,556	57,556	1
		**Mean**	**0.998786118**
		**SD**	**0.003129442**

**Table 5:** Frame count, the total number of actual eyeblinks in each sample and duration (in minute).

Sample File	Frame Count in the File	Count of Real Eyeblinks	Duration of the Video Sample
S1	31,071	21	4:18
S2	32,230	93	5:22
S3	41,388	61	6:00
S4	8720	19	1:12
S5	34,355	51	4:46
S6	20,460	64	2:50
S7	32,074	14	4:27
S8	11,278	8	1:33
S9	57,557	235	9:35
S10	41,684	169	6:56
S11	36,345	105	6:03
S12	43,289	193	8:09

**Table 6:** The performance evaluation of the proposed method.

GT	TP	FN	FP	DB	Precision	Recall	Accuracy	FDR
21	19	2	2	23	0.905	0.905	0.826	0.095
93	82	11	8	90	0.911	0.882	0.812	0.089
61	56	5	5	61	0.918	0.918	0.848	0.082
19	14	5	1	15	0.933	0.737	0.700	0.067
51	45	6	3	48	0.938	0.882	0.833	0.063
64	58	6	4	62	0.935	0.906	0.853	0.065
14	12	2	4	16	0.750	0.857	0.667	0.250
8	8	0	0	8	1.000	1.000	1.000	0.000
235	210	22	32	232	0.957	0.948	0.910	0.043
169	160	9	5	165	0.970	0.947	0.920	0.030
105	99	6	6	105	0.943	0.943	0.892	0.057
193	186	7	10	196	0.949	0.964	0.916	0.051
Mean:					0.918	0.904	0.839	0.082
Standard deviation:					0.060	0.063	0.088	0.060

**Figure A1:**
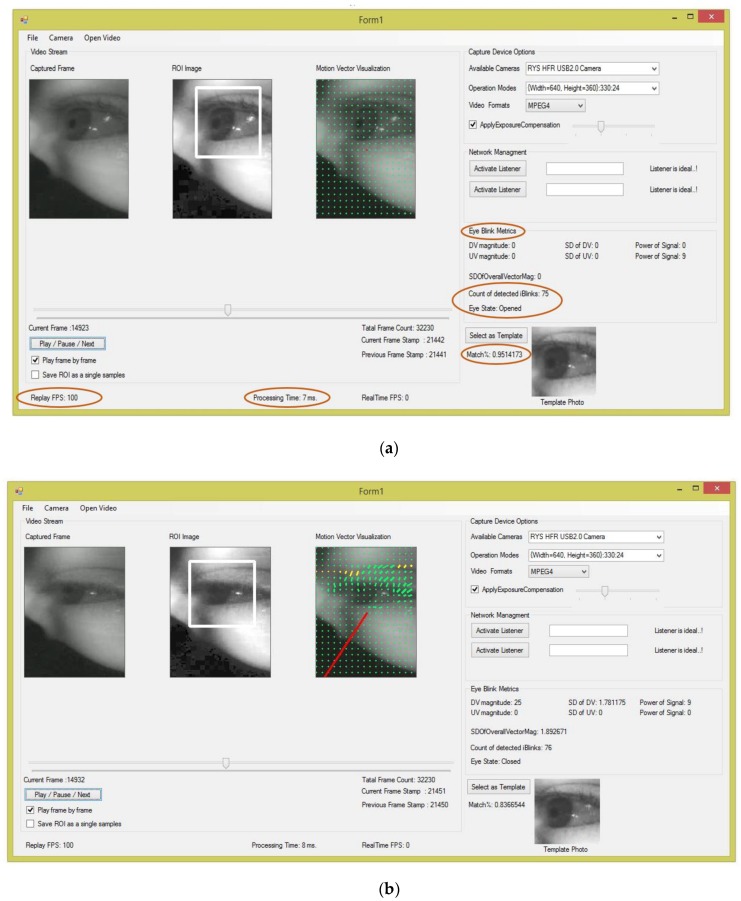

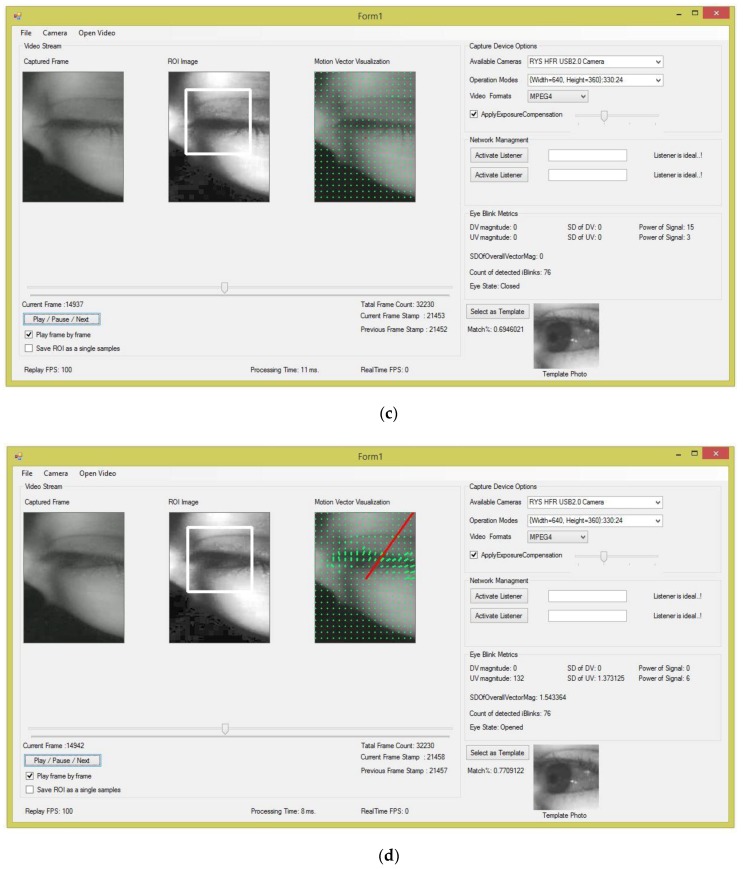
Eyeblink detection application in action: (**a**) when the eye is open; (**b**) during the closing phase; (**c**) during the pause phase; (**d**) during the reopening phase.

## Abbreviations

The following abbreviations have been used in this paper:

HMD	head-mounted display
VR	virtual reality
IVE	immersive virtual environment
EEG	electro-encephalography
EOG	electro-oculogram
FFBP	feed-forward backpropagation
SNR	signal-to-noise ratio
LDR	light-dependent resistor
FSM	finite state machine
FOV	field of view
FPS	frames per second
VE	virtual environment
ROI	region of interest
HCC	Haar cascade classifier
ROC	receiver operation characteristics
NCCC	normalised cross correlation coefficient
DMV	downward motion vector
UMV	upward motion vector
GT	ground truth
TP	true positive
FN	false negative
FP	false positive
DB	detected eyeblinks
FDR	false discovery rate

## Appendix A. Materials and Equipment

During this study, the following hardware equipment was used: IBM pc with intel core i7-7820HQ CPU at 3.6 GHz, system memory (RAM) 16 gigabyte and NVIDIA GeForce GTX780. For the display, the virtual environment HTC-Vive VR headset was utilised. For monitoring the eye inside the VR headset, a high-speed USB camera from *Kayeton^™^* with a resolution of 640_Width_ × 360_Height_ pixels at 120 fps capture capability was used. Regarding the software implementation during the study, Microsoft Visual Studio 2015 was used as programming API with Accord.net framework for image acquisition and processing. Unity game engine was used to implement the virtual environment. The implemented application employed to process the captured video stream localises the eye, identifies the eyeblinks and classifies the eye state.

The evaluation results will be sent by the application to the Unity game engine via an Ethernet socket using the home IP address (127.0.0.1). Figure A1 shows the proposed method that has been implemented with Microsoft Visual Studio to process the captured video stream in action. Figure A1 shows the GUI of the eyeblink detection application as well as some highlighted parameters, for instance, the template image, the matching percentage between the template image and the current detected ROI, eye state, count of detected eyeblinks and the processing time for the current frame. The application can record the camera video stream for developing and diagnoses purposes. Additionally, the GUI shows the visualisation of the motion vector and localising the ROI.
